# Leukocytes in Cerebral Thrombus Respond to Large-Vessel Occlusion in a Time-Dependent Manner and the Association of NETs With Collateral Flow

**DOI:** 10.3389/fimmu.2022.834562

**Published:** 2022-02-17

**Authors:** Xi Chen, Li Wang, Meiling Jiang, Lin Lin, Zhaojing Ba, Hao Tian, Guangjian Li, Lin Chen, Qu Liu, Xianhua Hou, Min Wu, Lu Liu, Wenying Ju, Wen Zeng, Zhenhua Zhou

**Affiliations:** ^1^ Department of Neurology, Southwest Hospital, Third Military Medical University (Army Medical University), Chongqing, China; ^2^ Department of Neurology, Zigong Third People’s Hospital, Zigong, China; ^3^ Department of Cell Biology, Third Military Medical University (Army Medical University), Chongqing, China; ^4^ State Key Laboratory of Trauma, Burn and Combined Injury, Chongqing, China

**Keywords:** thrombus, inflammation, ischemic stroke, time to reperfusion, collateral flow, NET

## Abstract

Thrombus components are dynamically influenced by local blood flow and blood immune cells. After a large-vessel occlusion stroke, changes in the cerebral thrombus are unclear. Here we assessed a total of 206 cerebral thrombi from patients with ischemic stroke undergoing endovascular thrombectomy. The thrombi were categorized by time to reperfusion of <4 h (T4), 4–8 h (T4–8), and >8 h (T8). The cellular compositions in thrombus were analyzed, and relevant clinical features were compared. Both white blood cells and neutrophils were increased and then decreased in thrombus with time to reperfusion, which were positively correlated with those in peripheral blood. The neutrophil extracellular trap (NET) content in thrombus was correlated with the degree of neurological impairment of patients. Moreover, with prolonged time to reperfusion, the patients showed a trend of better collateral grade, which was associated with a lower NET content in the thrombus. In conclusion, the present results reveal the relationship between time-related endovascular immune response and clinical symptoms post-stroke from the perspective of thrombus and peripheral blood. The time-related pathological changes of cerebral thrombus may not be the direct cause for the difficulty in thrombolysis and thrombectomy. A low NET content in thrombi indicates excellent collateral flow, which suggests that treatments targeting NETs in thrombi might be beneficial for early neurological protection.

## Introduction

Thrombus-based cerebrovascular occlusion is the main cause of acute ischemic stroke (AIS), which has a high rate of death and disability worldwide (1). Despite therapeutic improvements involving the use of intravenous thrombolysis and endovascular thrombectomy, these techniques are highly time dependent (2). Even for immunotherapy (3, 4), one of the promising targets for ischemic stroke, we find that it is important to take intervention time into account. In clinical practice, the consensus is that reducing the occlusion time of cerebral vessels promotes a successful endovascular recanalization and favorable neurological outcomes in ischemic stroke (5, 6). In addition to time to reperfusion, collateral flow status is another important prognostic factor, but few clinical studies have reported possible methods to effectively enhance early collateral flow (7). The sudden interruption of cerebral blood flow contributes to local blood cell traps, adhesion of platelets and leukocytes, and activation of the coagulation cascade (8). Given these facts, the quantification of thrombus components under different times of occlusion and different statuses of collateral flow would help clinicians better understand the dynamic pathological changes in thrombi after stroke onset, guide individualized recurrence prevention, and provide more evidence for revealing the mechanism of time-dependent thrombolysis resistance (9).

Much progress has been made in understanding the cellular components of thrombi in regard to different stroke etiologies (10, 11), treatment means (12, 13), and clinical features (14). Red blood cells (RBCs), fibrins, platelets, and white blood cells (WBCs) are the main cell components of thrombi, and the relative content of each provides much information about the stability of the thrombus (9) and its sensitivity to antithrombotic agents (15). Neutrophil extracellular traps (NETs), released by activated neutrophils, have been found to be the key factors in thrombolysis resistance (13) and have recently been proven to be associated with the clinical outcomes of patients with AIS (16). In fact, NET formation in thrombi directly affects thrombus stability (17) and aggravates the difficulty of thrombolysis (13) and thrombectomy (18). However, the dynamic changes of these components during the occlusion of a blood vessel remain obscure.

Therefore, to enhance our understanding of the characteristics of cerebral thrombi with prolonged time to reperfusion, we used Martius Scarlett Blue (MSB) and immunohistochemical staining to distinguish the different compositions of thrombus sections and collected homologous hematological parameters to investigate the association between thrombi and peripheral blood. Further, we identified the content of NETs, which have been linked to thrombosis and vascular remodeling (19, 20), in different thrombus sections. In addition, we evaluated the pretreatment collateral flow as the time to reperfusion extended and the correlation between collateral status and NETs. Here, we report on the time-dependent alteration of cerebral thrombus composition and its correlation to collateral flow after the occurrence of stroke.

## Materials and Methods

### Patient Selection and Clinical Data

The data related to this study are available from the corresponding author upon reasonable request. The study was approved by the Ethics Committee of the First Affiliated Hospital of Army Medical University [(A)KY2021023] and the Ethics Committee of the Zigong Third People’s Hospital (2021–01–01). Informed consent was obtained from the patients. Thrombi from patients with AIS from Southwest Hospital, Third Military Medical University, and Zigong Third People’s Hospital who underwent thrombectomy between December 2017 and November 2020 were consecutively included.

We included 235 patients who fulfill the following criteria: (1) clinical indication for mechanical thrombectomy of acute large-vessel occlusion and (2) successfully retrieved thrombi for histological analysis. Endovascular thrombectomy was performed according to the relevant guidelines. After the procedure, the thrombi were retrieved, immediately fixed in 4% paraformaldehyde, and then embedded in paraffin for histological analysis. In total, 235 thrombi were stained; 15 thrombi, due to time to reperfusion lasted for more than 24 h, and 14 thrombi, due to missing clinical data, were excluded. Patients with previous use of immunomodulatory drugs were not included. The flow diagram of the inclusion of thrombi was presented in **Supplementary Figure S1**.

The following clinical data were collected: time from symptom onset to reperfusion, occlusion site, stroke causes according to the international Trial of Org 10172 in Acute Stroke Treatment (TOAST) classification (21), procedural technique, procedural time, modified thrombolysis in Cerebral infarction (mTICI) score after recanalization, pre-interventional National Institutes of Health Stroke Scale (NIHSS) score, NIHSS at discharge, modified Rankin Scale (mRS) scores within 90 days, antithrombotic treatment (application of antithrombotic drugs, such as clopidogrel or aspirin, at stroke onset), thrombolytic treatment [application of intravenous tissue-type plasminogen activator (rt-PA)], and number of retraction maneuvers.

The TOAST classification, mTICI scores, and NIHSS scores on admission and at discharge were assessed by a neurologist. The mRS scores were obtained by phone interview.

The median and interquartile range (IQR) for time from symptom onset to time to reperfusion was 329.5 (262.5–435.0) min. To investigate the effect of time from symptom onset to reperfusion on the composition of AIS thrombi, the patients were divided into three groups: <4 h (T4 group, *n* = 40), 4–8 h (T4–8 group, *n* = 122), and >8 h (T8 group, *n* = 44). The pre-thrombectomy hematological parameters, including complete blood counts, fibrin, international normalized ratio (INR), and D-dimer, were assessed based on time to blood collection.

### Histology and Immunohistochemistry

Thrombi embedded in paraffin were cut along the longest plane into 5-μm-thick sections. Before histological and immunohistochemical staining, the sections were mounted on slides and deparaffinized by immersion in xylene, rehydrated in progressively reduced concentrations of ethanol, and washed in phosphate-buffered saline. The observer was completely blinded to the classification.

MSB staining was conducted according to the manufacturer’s instructions (Solarbio, Beijing Solarbio Science and Technology Co.). The stained thrombus sections were photographed (magnification, ×40) under a ZEISS microscope. Representative MSB-stained slides were sent for whole-slide scanning using Olympus VS200.

For immunohistochemical staining, antigen retrieval was performed with citrate-EDTA buffer in 95°C water bath. Then, the sections were blocked with 10% goat serum in phosphate-buffered saline + 0.1%Triton X-100 and incubated with primary antibody at 4°C for two nights. Next, the sections were washed and then incubated with the secondary antibody. The antibodies used were rabbit anti-MPO (1:200, Proteintech), mouse anti-MPO (1:100, Proteintech), rabbit anti-histone H4 (citrulline 3) (1:200, Millipore), Alexa Fluor 488 goat anti-rabbit IgG, Alexa Fluor 568 goat anti-rabbit IgG, and Alexa Fluor 568 goat anti-mouse IgG (all 1:1,000, Invitrogen). All fluorescent images of the mounted sections were captured with an Olympus SpinSR1 confocal microscope.

Areas that were positive for MSB or immunohistochemical staining were calculated from an average of 5- fields/sample with ImageJ 1.37v software. Cellular components (RBCs, fibrin/platelets, and WBCs) in MSB-stained sections were distinguished using the color deconvolution program in ImageJ, and the percentage of each component relative to the whole thrombus was calculated.

### Collateral Flow Assessment

Digital subtraction angiography (DSA)-based collateral grading was performed using the American Society of Interventional and Therapeutic Neuroradiology/Society of Interventional Radiology (ASITN/SIR) Collateral Flow Grading System for pretreatment DSA (22). In this study, the pre-interventional collateral flow of patients with anterior circulation occlusion was categorized as poor (Higashida score, 0–1), intermediate (Higashida score, 2), good (Higashida score, 3), and excellent (Higashida score, 4) (23). All raters, who were blinded to the clinical findings, assessed the DSA results independently and resolved disagreements by consensus.

### Statistical Analysis

Categorical variables were compared using the chi-square test. For continuous variables, normality and equal variances between group samples were assessed using the Shapiro–Wilk normality test and homogeneity of variance test, respectively. When normality and equal variance between sample groups were achieved, one-way ANOVA (followed by Bonferroni’s multiple-comparisons test or Dunnett’s T3 test) was used. Where normality or equal variance between samples failed, Kruskal–Wallis one-way ANOVA (followed by Bonferroni’s correction) or Mann–Whitney *U*-test was performed. Data are presented as the mean ± standard deviation, median (IQR), or number (%). Only 4 of the study variables had missing values: NIHSS score (post) had 3 missing values, fibrinogen (g/L) had 1 missing value, INR (s) had 2 missing values, and D-dimer (mg/L) had 2 missing values. The missing values (<1.5%) were imputed with regression multiple imputation method. Pearson correlation or Spearman correlation was adopted for the correlation analysis. To investigate the association between multiple independent variables and WBCs % in thrombi, multiple linear regression was conducted. After the univariate analysis, the investigators performed a multivariable logistic regression, adjusting for the following prespecified confounders: age, men, coronary artery disease, atrial fibrillation, stroke cause (TOAST), procedure time, NIHSS score (pre), and NIHSS (post). All statistical analyses were performed using IBM SPSS Statistics 22 (IBM Software, Chicago, IL, USA). We considered a value of *p <*0.05 to indicate statistical significance.

## Results

### Study Population

A total of 206 thrombi from AIS patients (mean age: 74.0, 66.0–81.0 years; men, 45.6%) were analyzed in this study. The median (IQR) time to reperfusion from symptom onset to reperfusion was 191.0 (135.0–215.8) min in T4, 326.5 (279.5–397.3) min in T4–8, and 574.0 (517.8–731.3) min in T8. The clinical characteristics of the patients are presented in **Table 1**. In brief, a higher percentage of patients in T4 (*P* < 0.001) presented with atrial fibrillation in their medical history than patients in the T4–8 and T8 groups. In the T4 group, less patients were classified as arterioembolic (TOAST-1) (*P* < 0.001), while more patients were classified as cardioembolic (TOAST-2) (*P* < 0.001). Regarding other clinical factors, the T8 group had less thrombolytic treatment (*P* < 0.001), lower stentriever rate (*P* = 0.009), longer procedural time (*P* < 0.001), smaller number of maneuvers (*P* = 0.004), and lower NIHSS score (pre) (*P* < 0.001) than the T4 and T4–8 groups.

**Table 1:** Characteristics of all patients.

	Total (*n* = 206)	T4 (*n* = 40)	T4–8 (*n* = 122)	T8 (*n* = 44)	*P*-value
Age, years; median (IQR)	74.0 (66.0–81.0)	75.5 (66.3–81.0)	74.0 (66.0–81.3)	66.5 (62.0–79.8)	0.143
Male (%)	94 (45.6)	13 (32.5)	59 (48.4)	22 (50.0)	0.175
Medical history (%)					
Hypertension	107 (51.9)	18 (45.0)	64 (52.5)	25 (56.8)	0.548
Diabetes	37 (18.0)	6 (15.0)	22 (18.0)	9 (20.5)	0.809
Hyperlipidemia	22 (11.2)	4 (10.0)	11 (9.0)	7 (15.9)	0.442
Coronary artery disease	74 (35.9)	15 (37.5)	49 (40.2)	10 (22.7)	0.115
Active smoker	26 (12.6)	3 (7.5)	16 (13.1)	7 (14.9)	0.494
Atrial fibrillation	112 (54.4)	29 (72.5)	70 (57.4)	13 (29.5)	<0.001*
Prior ischemic stroke	27 (13.1)	4 (10.0)	18 (14.8)	5 (11.4)	0.688
Prior hemorrhagic stroke	2 (1.0)	0 (0)	1 (0.8)	1 (2.2)	0.550
Clinical data					
Time to reperfusion, min	329.5 (262.5–435.0)	191.0 (135.0–215.8)	326.5 (279.5–397.3)	574.0 (517.8–731.3)	<0.001*
Occlusion site (%)					
Anterior circulation	184 (89.3)	37 (92.5)	111 (90.9)	36 (81.8)	0.185
Posterior circulation	22 (10.7)	3 (7.5)	11 (9.0)	8 (18.2)	0.185
Stroke cause (TOAST) (%)					
Arterioembolic	38 (18.4)	1 (2.5)	18 (14.8)	19 (43.2)	<0.001*
Cardioembolic	155 (75.2)	38 (95.0)	95 (77.9)	22 (50.0)	<0.001*
Cryptogenic	13 (10.7)	1 (2.5)	9 (7.3)	3 (6.8)	0.539
Antithrombotic treatment (%)	27 (13.1)	6 (15.0)	19 (15.6)	2 (5.0)	0.165
Thrombolytic treatment (%)	71 (34.5)	23 (57.5)	45 (36.9)	3 (6.8)	<0.001*
Procedural technique (%)					
Stentriever	196 (95.1)	39 (97.5)	119 (97.5)	38 (86.4)	0.009*
Direct aspiration	7 (3.4)	1 (2.5)	2 (1.6)	4 (9.1)	0.061
Arterial thrombolysis	38 (18.4)	11 (27.5)	22 (18.0)	5 (11.4)	0.160
Balloon angioplasty	15 (7.3)	0 (0.0)	10 (8.2)	5 (11.4)	0.112
ADAPT	5 (2.4)	0 (0)	2 (1.6)	3 (6.8)	0.086
Stent placement	15 (7.3)	0 (0)	9 (7.4)	6 (13.6)	0.056
Procedure time, min	79.0 (55.0–112.8)	59.0 (40.0–73.0)	81.0 (60.0–110.5)	108.5 (68.0–147.5)	<0.001*
Number of maneuvers	1.0 (1.0–2.0)	1.0 (1.0–2.0)	2.0 (1.0–3.0)	1.0 (1.0–2.0)	0.004*
mTICI score 2b-3 (%)	198 (96.1)	39 (97.5)	119 (97.5)	40 (90.9)	0.131
NIHSS score (pre)	20.0 (15.8-24.0)	21.5(19.0-25.0)	21.0 (16.0-25.0)	15.0 (11.0-19.8)	<0.001*
NIHSS score (post)	7.0 (2.0–12.1)	3.0 (1.0–11.3)	8.0 (3.0–14.0)	6.5 (2.0–10.0)	0.037*
mRS-90d 0–2 (%)	109 (52.9)	24 (60.0)	62 (50.8)	23 (52.3)	0.598

ADAPT, a direct aspiration first-pass technique; mTICI, modified thrombolysis in cerebral infarction; NIHSS, National Institutes of Health Stroke Scale; mRS, modified Rankin Scale; RBCs, red blood cells; WBCs, white blood cells. “*” represents “P < 0.05”.

### Thrombus Composition at Different Times to Reperfusion

**Figure 1:**
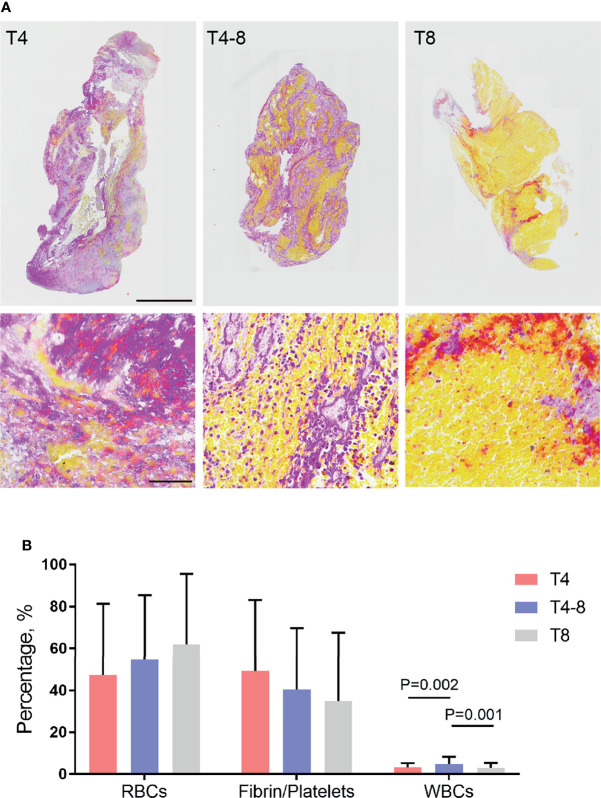
Red blood cells (RBCs), fibrin/platelets, and white blood cells (WBCs) in thrombi in relation to the different durations of time to reperfusion. **(A)** Representative image of thrombi sections comparing <4 h (T4), 4–8 h (T4–8), and >8 h (T8) time to reperfusion. Martius Scarlett Blue staining was used to detect RBCs (yellow), fibrin/platelets (red or purple), and WBCs (blue). Low-magnification scale bar = 1 mm; high-magnification scale bar = 60 μm. **(B)** Box plot indicating thrombus composition changes with different times to reperfusion. The percentage of each component (RBCs, fibrin/platelets, and WBCs) relative to the whole thrombus was calculated.

All thrombi were stained with MSB to demonstrate the presence of RBCs (yellow), fibrin/platelets (red or purple), and WBCs (blue) (**Figure 1A**). The histological pictures of the three groups are shown in **Figure 1A**. The morphological characterization of thrombi showed that the elapsed time to reperfusion had a effect on RBC, fibrin/platelets, and WBC composition of thrombi (**Supplementary Table S1**). Specifically, the WBC composition of thrombi was significantly higher in the T4–8 group than in the T4 group (*P* = 0.002) and T8 group (*P* = 0.001) (**Figure 1B**). In addition, there were no significant changes in RBC (*P* = 0.059) and fibrin/platelet (0.055) composition of thrombi among the three groups (**Figure 1B**). There was an independent association between WBC composition in thrombi and time to reperfusion (**Supplementary Table S2**).

**Figure 2:**
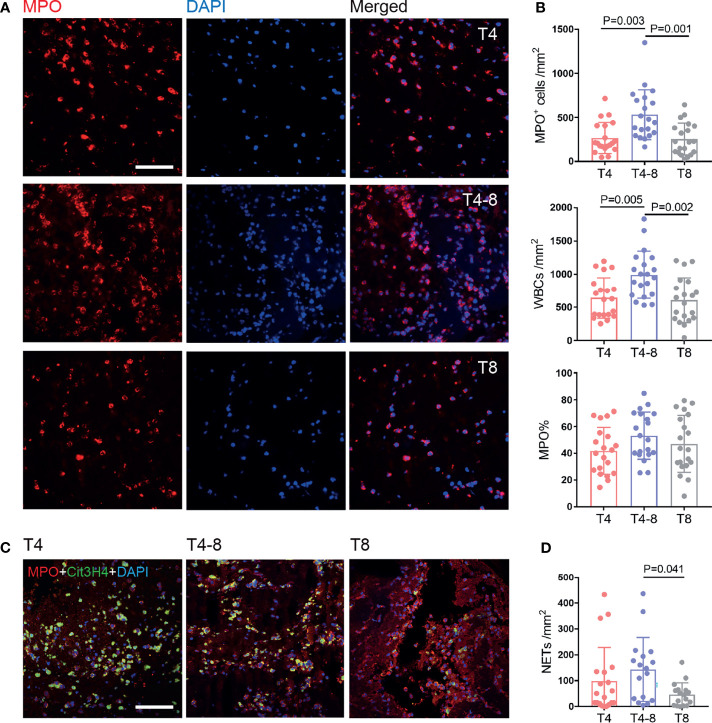
Neutrophil and NET changes in thrombi at different times after reperfusion. **(A)** Representative immunohistochemical illustration of neutrophils (MPO, red) in thrombi of the T4, T4–8, and T8 groups. The nuclei are counterstained with DAPI. Scale bar = 50 μm. **(B)** Quantification of MPO+ cells/mm^2^ (top), white blood cells/mm^2^ (middle), and MPO% (bottom) in thrombi according to different times to reperfusion. *N* = 20/group. **(C)** Representative immunohistochemical illustration of NETs in thrombi by staining for MPO (red) and Cit3H4 (green). The nuclei are counterstained with DAPI. Scale bar = 50 μm. **(D)** Quantification of NETs/mm^2^ in thrombi according to time to reperfusion. T4, *n* = 20; T4–8, *n* = 17; T8, *n* = 17. NETs, neutrophil extracellular traps.

To indicate thrombi composition changes as time to reperfusion, we generated a line graph for RBCs, fibrin/platelets, and WBCs (**Supplementary Figure S2A**). The WBC composition was obviously elevated and then decreased with time to reperfusion (*P* = 0.019) (**Supplementary Figure S2B**). We then further focused on the levels of neutrophils and NETs in thrombi. The results showed that the neutrophils were rapidly recruited to the thrombus and were observable at T4, remained high at T4–8 (*P* = 0.003), and decreased at T8 (*P* = 0.001), which was consistent with the WBC findings (**Figures 2A, B**). Moreover, the percentage of neutrophils (neutrophil%) was predominant in thrombi at T4 (41.7 ± 17.6%), T4–8 (53.08 ± 17.6%), and T8 (46.9 ± 21.2%), but there was no significant difference among them (*P* = 0.173). Interestingly, NETs were generated quickly in the early stages (T4 and T4–8) and reduced in the late stage (T8, *P* = 0.041) (**Figures 2C, D**).

### Peripheral Blood Composition at Different Times to Reperfusion

To identify peripheral immune responses as time to reperfusion increased, we assessed the following parameters in peripheral blood before the endovascular thrombectomy (**Table 2**). As previously reported, the WBCs increased with prolonged ischemia duration (*P* = 0.009). Platelets in peripheral blood in the T4–8 group were increased compared with those in the T4 group (*P* = 0.018), which was different from the thrombus findings.

**Table 2:** Changes in the peripheral blood of all patients.

	Total (*n* = 182)	T4 (*n* = 90)	T4–8 (*n* = 62)	T8 (*n* = 30)	*P*-value
WBCs (10^9^/L)	7.9 (6.3–10.0)	7.2 (5.9–8.9)	8.3 (6.4–11.1)	8.9 (6.8–10.6)	0.009*
RBCs (10^12^/L)	4.2 (3.9–4.6)	4.2 (3.8–4.6)	4.1 (3.9–4.5)	4.2 (3.9–4.6)	0.941
Platelets (10^9^/L)	145.5 (116.0–189.3)	129.5 (108.8–182.3)	159.0 (126.8–217.3)	150.0 (117.8–188.8)	0.016*
Fibrinogen (g/L)	2.7 (2.4–3.3)	2.7 (2.4–3.1)	2.7 (2.3–3.3)	3.0 (2.6–3.5)	0.017
Neutrophil, %	78.7 (70.6–84.4)	75.1 (64.3–81.2)	81.9 (74.1–87.1)	81.6 (74.1–85.4)	<0.001*
Lymphocyte, %	14.7 (10.7–21.3)	17.3 (12.5–26.5)	12.8 (8.3–17.5)	13.0 (8.3–18.6)	<0.001*
Monocyte, %	5.3 (4.0–6.7)	5.7 (4.1–7.2)	4.6 (3.7–6.2)	5.5 (4.1–6.6)	0.041*
Neutrophils (10^9^/L)	5.8 (4.3–8.0)	5.2 (3.9–6.7)	7.0 (4.5–9.0)	7.1 (5.3–8.8)	0.001*
Lymphocytes (10^9^/L)	1.2 (0.8–1.6)	1.3 (1.0–1.7)	0.9 (0.7–1.4)	1.2 (0.8–1.4)	0.020*
Monocytes (10^9^/L)	0.4 (0.3–0.5)	0.4 (0.3–0.5)	0.4 (0.3–0.5)	0.5 (0.4–0.6)	0.225
INR (s)	1.1 (1.0–1.1)	1.1 (1.0–1.1)	1.0 (1.0–1.1)	1.0 (0.9–1.1)	0.468
D-dimer (mg/L)	0.8 (0.5–1.7)	0.8 (0.4–1.7)	0.8 (0.5–2.0)	0.8 (0.4–2.0)	0.812

WBCs, white blood cells; RBCs, red blood cells; LDL, low-density lipoprotein. “*” represents “P < 0.05”.

Moreover, the neutrophil% in the T4 group was significantly lower than that in the T4–8 (*P* < 0.001) and T8 (*P* < 0.001) groups. The neutrophils were lower in the T4 group than in the T4–8 (*P* = 0.029) and T8 (*P* = 0.003) groups. Conversely, the T4 group had a greater lymphocyte% than the T4–8 (*P* < 0.001) and T8 (*P* < 0.001) groups. There were more lymphocytes in the T4 group than in the T4–8 group (*P* = 0.037). Furthermore, the monocyte% in the T4 group was obviously higher than in the T4–8 group (*P* = 0.038). However, there were no significant differences in RBCs, monocytes, INR, and D-dimer among the three groups.

### NETs in Thrombi Were Associated With the Clinical Features of the Patients

**Figure 3:**
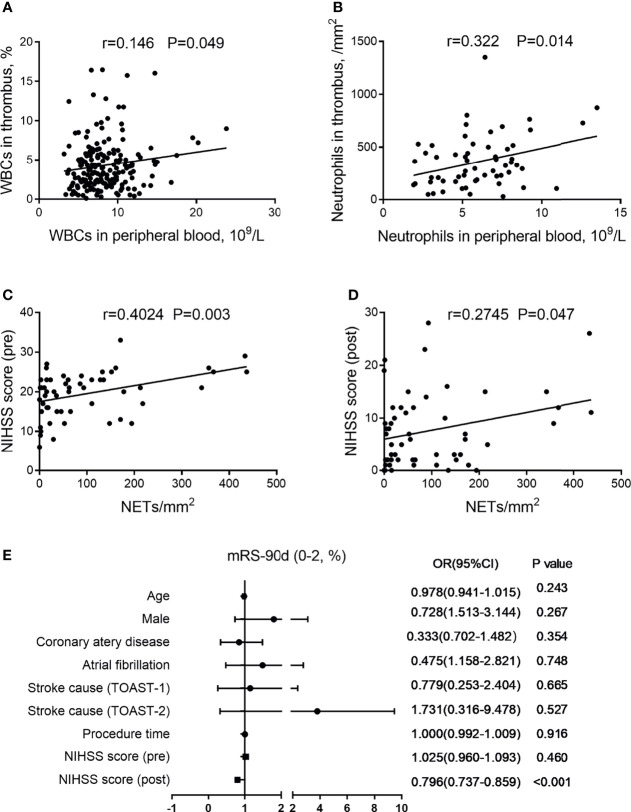
Association of (neutrophil extracellular traps) NETs in thrombi with clinical features. **(A)** Correlation between white blood cells (WBCs) in thrombi and WBCs in peripheral blood. **(B)** Correlation between neutrophils in thrombi and neutrophils in peripheral blood. **(C)** Correlation between NIHSS score (pre) and NETs in thrombi. **(D)** Correlation between NIHSS score (post) and NETs in thrombi. **(E)** Forest plot of multivariate logistic regression analysis for patients with AIS, including possible outcome-influencing factors regarding the correlation analysis. AIS, acute ischemic stroke; NIHSS, National Institutes of Health Stroke Scale; mRS, modified Rankin Scale.

An increased WBC count in thrombi as time to reperfusion was significantly correlated with the elevated WBCs in peripheral blood (*r* = 0.146, *P* = 0.049; **Figure 3A**). The neutrophil counts in thrombi were likewise strongly correlated with those in peripheral blood (*r* = 0.322, *P* = 0.014; **Figure 3B**).

Since WBC-mediated immune responses in both thrombi and peripheral blood may affect neurological function *via* an impaired blood–brain barrier, we found that NETs in thrombi were positively related to the NIHSS score (pre) (*r* = 0.4024, *P* = 0.003; **Figure 3C**) and NIHSS score (post) (*r* = 0.2745, *P* = 0.047; **Figure 3D**). Moreover, we performed a multivariable logistic regression analysis for patients with AIS that included possible confounders [age, male, coronary artery disease, atrial fibrillation, stroke cause, procedure time, NIHSS score (pre), and NIHSS score (post)] (**Figure 3E**). Among them, we found that NIHSS score (post) was an independent factor associated with clinical outcome.

### NETs in Thrombi Were Associated With the Collateral Status in Patients

**Figure 4:**
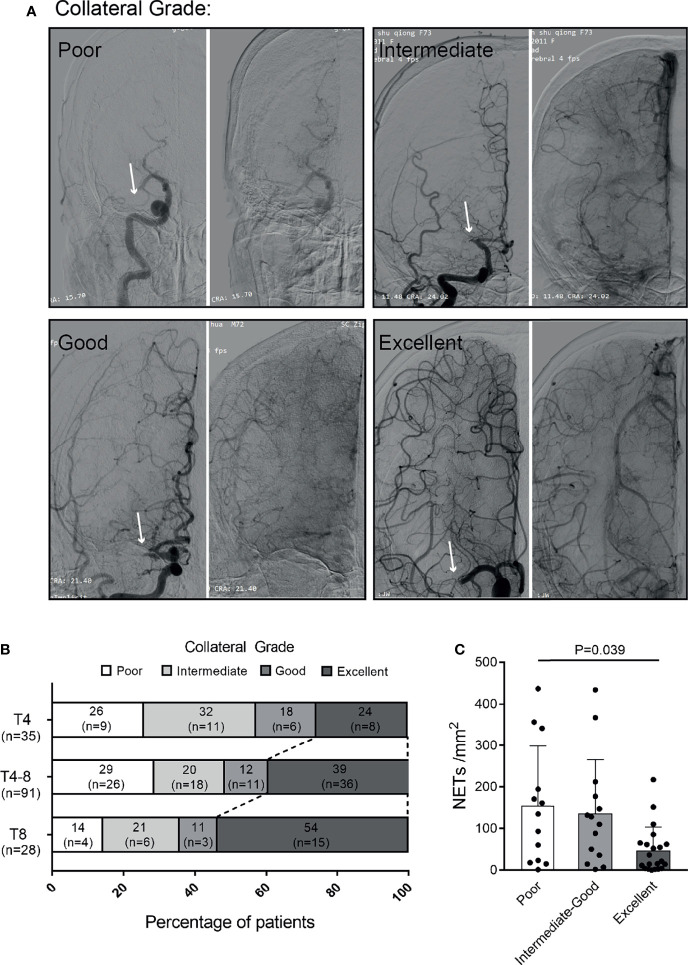
Association of NETs in thrombi with collateral flow of patients with ischemic stroke. **(A)** Examples of collateral flow with digital subtraction angiography (DSA)-based collateral grades. All DSA images show proximal middle cerebral artery (MCA) occlusions (arrows). The DSA consists of arterial (left panel) and venous phases (right panel). Poor grade: no collaterals visible or slow collaterals visible only in the late phase. Intermediate grade: rapid collateral flow to part of the occluded MCA territory with persistence of some of the defect. Good grade: slow but complete collateral flow in the occluded MCA territory. Excellent grade: rapid and complete collateral flow in the occluded MCA territory. **(B)** Distribution of collateral grade in patients with different times to reperfusion. **(C)** Quantification of NETs/mm^2^ in the thrombi of patients with poor (*n* = 13), intermediate to good (*n* = 14), and excellent (*n* = 21) collateral flows. NETs, neutrophil extracellular traps.

To assess whether NET formation affected the clinical symptoms by impairing the collateral flow, we further compared the NET contents of thrombi among patients with different collateral grades. Examples of DSA-based collateral grades are given (**Figure 4A**). A longer time to reperfusion was significantly associated with a higher collateral grade (*P* = 0.038) (**Figure 4B**). Moreover, the NET content in thrombi was decreased in patients with excellent collateral flow compared to those with a poor one (*P* = 0.039) (**Figure 4C**).

## Discussion

In this study, we identified the evolution of thrombus composition under time to reperfusion of <4, 4–8, and >8 h. As a main finding, the leukocytes and neutrophils in thrombi were accumulated in a time-dependent manner, which were consistent with those in peripheral blood. The thrombi of patients with over 8 h of occlusion were found to be NET-poor, which was associated with a better collateral flow and milder clinical symptom. Nearly all thrombi contain NETs (24), and targeting NETs accelerates rt-PA-based thrombolysis (13). Here our findings provide new evidence that NETs might be a vital factor that affects collateral flow status at stroke onset.

We demonstrate that as time to reperfusion prolongs, there are dynamic changes in the amount of WBCs in thrombi. As reported, the infiltration of WBCs in brain can cause differences in blood–brain barrier damage (25), brain function injury (26), and post-stroke angiogenesis (27). Although WBCs make up a small fraction of blood clots, they play a major role in the early stage after a stroke. Furthermore, time-dependent immune responses in thrombi and peripheral blood may interact as both the cause and effect (28). The dynamic transformation of hematological components may affect the composition of thrombi, while the sustained thromboembolism of cerebral vessels further mobilizes immune cells (29). Moreover, we assessed both thrombus composition and peripheral blood parameters, revealing the similarities and differences between them following stroke onset.

NETs, which are derived from the dominant WBC type, have attracted our attention because of their strong relation to clinical features. In line with a previous study (16), the NET content in thrombi was positively correlated with the NIHSS score at discharge, which is a predictive factor for favorable clinical outcomes in ischemic stroke. This phenomenon may be due to the neurotoxicity induced by protease release and degranulation of transmigrated neutrophils associated with NET formation (30). In addition, the decrease of NETs in thrombi may be related to the internalization and degradation function of macrophages (31). It has been demonstrated that perfusion image-based thrombolysis can extend thrombolysis to 4.5–9 h (32). The extension of time-window requires consideration of the safety and efficacy of the treatment, like the hemorrhagic risk, penumbra area, and thrombus properties. Hence, a reduction in NETs and fibrin/platelets in thrombi with a long occlusion time provides histological evidence for the possibility of prolonging the thrombolysis time-window.

Recent insights have suggested that NET formation impairs revascularization and vascular remodeling post-stroke (20). Kartika R et al. (19) found that NETs were prominent in the early stage of thrombosis. Analogously, our study shows that NETs were mostly present in the T4 and T4–8 groups and relatively low in the T8 group; this trend is consistent with the NIHSS score prethrombectomy. This time-dependent reduction in clinical symptoms could be explained by better pretreatment collateral status. Our study suggests that the recruitment of leukocytes to thrombi may affect neurological functions by not only releasing inflammatory factors and infiltrating the brain but also impairing collateral flow to occluded territories. The effect of NET contents in thrombi on collateral flow may be relevant to the different fluid shear stresses after occlusion, which initiates collateral remodeling *via* mechanoreceptors on endothelial cells (33). Notably, the timing for early intervention of WBCs or NETs in thrombi is quite important (34) when the recruitment of WBCs is relatively low. However, the mechanism underlying this process needs further proof in basic research.

Nevertheless, there are some limitations in this study. First, this work lacks the additional molecular evidence to combine the dynamic changes in thrombi with neurologic dysfunction post-stroke. Additionally, it is necessary to distinguish additional subtypes of leukocytes. It would be more precise if we could obtain the peripheral blood composition at the time of thrombus removal. Moreover, the process of thrombosis *in situ* is ignored.

Taken together, this study provides evidence about the dynamic progression of inflammatory responses both in thrombus and peripheral blood after a stroke happens. Among them, the NET levels in ischemic stroke thrombi are associated with pretreatment collateral status, which affects the neurological functions of patients. These findings suggest that early targeting of NETs may be a promising strategy for early protection of the brain after ischemic stroke occurs.

## Data Availability Statement

The original contributions presented in the study are included in the article/**Supplementary Material**. Further inquiries can be directed to the corresponding authors.

## Ethics Statement

The studies involving human participants were reviewed and approved by the Ethics Committee of the First Affiliated Hospital of Army Medical University [(A)KY2021023] and the Ethics Committee of the Zigong Third People’s Hospital (2021-01-01). The patients/participants provided their written informed consent to participate in this study.

## Author Contributions

ZZ and WZ contributed to the conception, supervision, and design of this article. XC, LW, GL, QL, LC, XH, LuL, MW, and WJ performed the experiments and data acquisition. XC, MJ, LiL, ZB, and HT contributed to data analysis. XC, WZ, and ZZ took charge of manuscript drafting and creation of figures. All authors contributed to the article and approved the submitted version.

## Funding

This work was supported by the National Key Research and Development Plan Young Scientists Program (no. 2017YFA0106000), the National Natural Science Foundation of China (nos. 81971130 and 81471194), the National Science Fund for Outstanding Young Scholars (no. 31822021), and Chongqing Technology Innovation and Application Development Program (no. cstc2019jscx-gksbX0064).

## Conflict of Interest

The authors declare that the research was conducted in the absence of any commercial or financial relationships that could be construed as a potential conflict of interest.

## Publisher’s Note

All claims expressed in this article are solely those of the authors and do not necessarily represent those of their affiliated organizations, or those of the publisher, the editors and the reviewers. Any product that may be evaluated in this article, or claim that may be made by its manufacturer, is not guaranteed or endorsed by the publisher.
