# Supplementary material for: Leukocytes in Cerebral Thrombus Respond to Large-Vessel Occlusion in a Time-Dependent Manner and the Association of NETs With Collateral Flow
Source: Front Immunol. 2022 Feb 17;13:834562. doi: 10.3389/fimmu.2022.834562 (PMC8891436; doi:10.3389/fimmu.2022.834562)
Supplement: Supplementary file 1 [file DataSheet_1.docx]

Supplementary Material

# Supplementary Data

Thrombectomy procedure

Mechanical thrombectomy was performed by experienced neurologists in accordance with local guidelines. All patients underwent Non-Contrast Computed Tomography imaging prior to the treatment to exclude intracranial hemorrhage. Intravenous rt-PA was administered to eligible patients based on clinical and imaging findings. Stent retriever, penumbra aspiration device or combination techniques were used in the procedure. The occlusion was reached using a guide catheter inserted via the femoral artery in most cases. After each pass, the device, intermediate catheter if withdrawn, and aspiration syringe were inspected for the presence of thrombus fragments. The device was gently washed with heparinised saline to remove thrombus fragments.

Digital subtraction angiography

DSA consists of arterial, capillary, and venous phases. Leptomeningeal collateral flow in the lesional hemisphere is usually seen until the late venous phase, when the normal circulation of the contralesional hemisphere is already washed out.

# Supplementary Figures and Tables

## Supplementary Figures


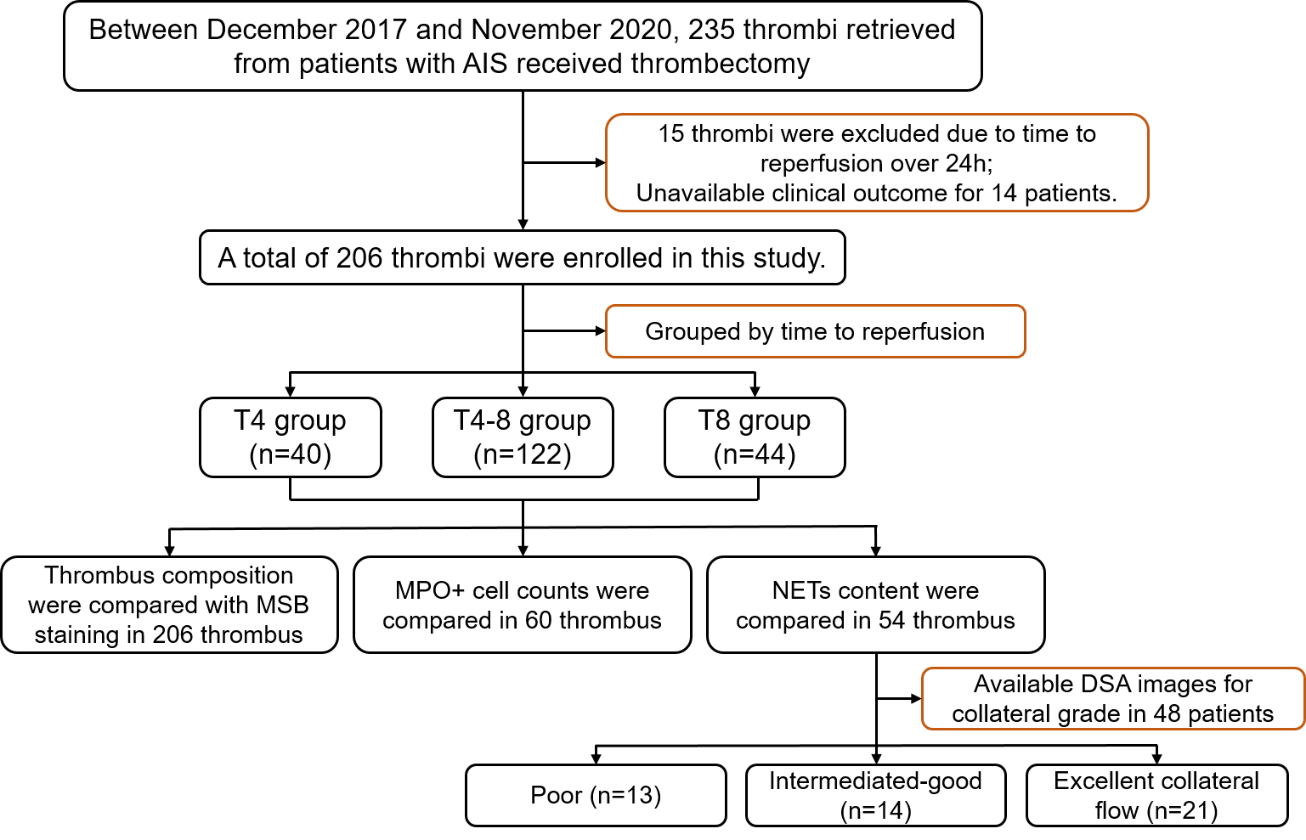


**Supplementary Figure 1.** Flow diagram of the inclusion of thrombi from patients with AIS in this study. AIS: acute ischemic stroke.


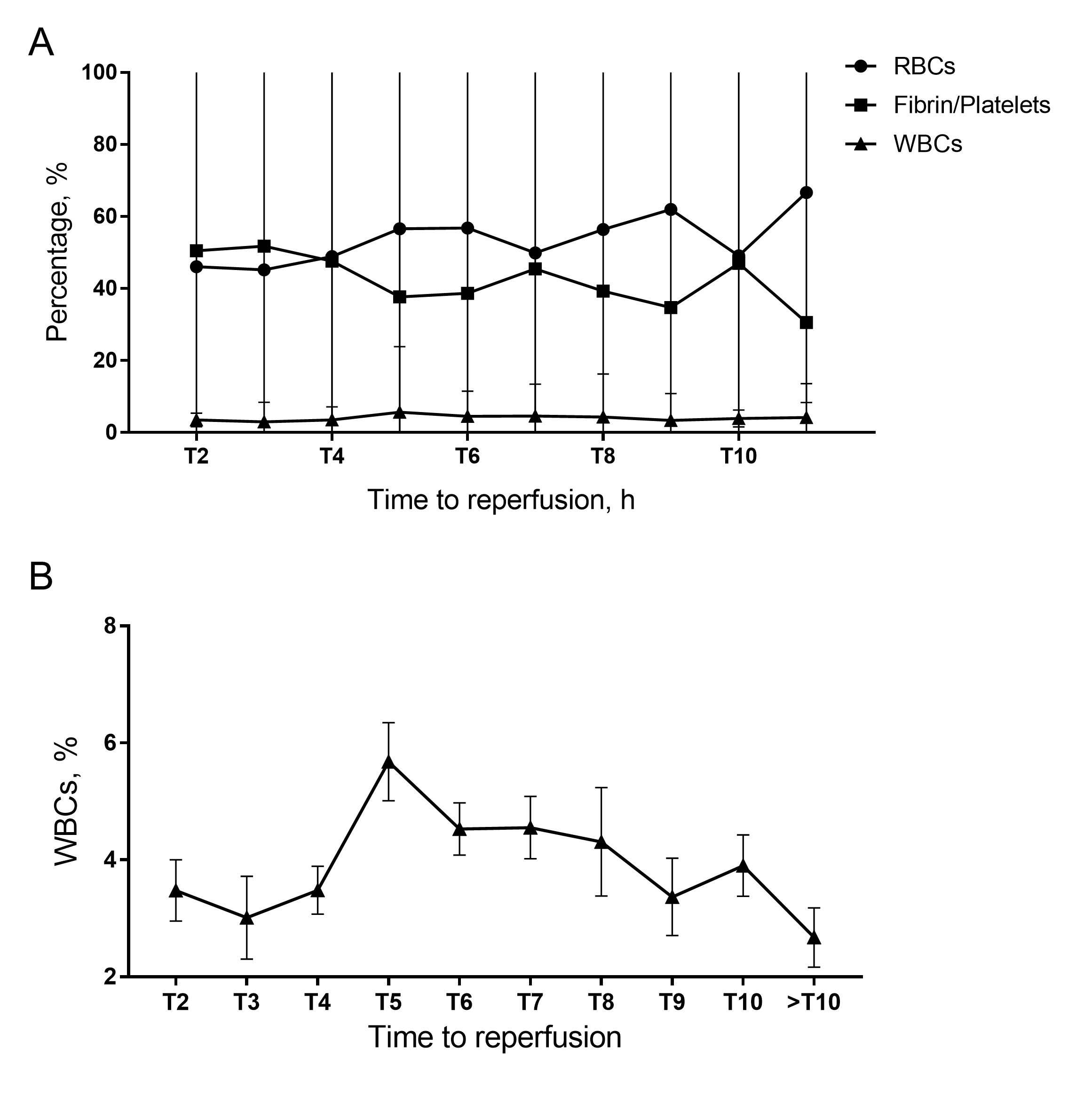


**Supplementary Figure 2.** Line graph illustrating thrombi composition changes with time to reperfusion prolongs. (A) RBCs, fibrin/platelets and WBCs composition of total 206 thrombi retrieved under different time to reperfusion. (B) WBCs accumulating in thrombi with time to reperfusion prolongs.

## Supplementary Tables

**Supplementary Table 1.** Thrombus composition of all patients.

| Thrombi composition | Total (n=206) | T4 (n=40) | T4-8 (n=122) | T8 (n=44) | P value |
| --- | --- | --- | --- | --- | --- |
| RBCs (%) | 57.1  (27.6-86.3) | 49.9  (13.9-82.6) | 56.7  (30.9-84.4) | 69.9  (36.4-93.0) | 0.059 |
| Fibrin/platelets (%) | 37.6  (9.6-65.8) | 45.2  (15.0-82.4) | 38.3  (12.3-63.3) | 24.4  (5.0-59.9) | 0.055 |
| WBCs (%) | 3.5 (2.1-5.6) | 3.1(2.0-4.6) | 4.2 (2.4-6.0) | 2.4 (1.2-4.7) | <0.001* |
|  |  |  |  |  |  |

**Supplementary Table 2.** Estimates of WBCs% and independent variables in multiple linear regression with significance.

| Parameter | Estimate | 95%CI  Lower Bound | 95%CI  Upper Bound | Standard Error | Significance level  P Value |
| --- | --- | --- | --- | --- | --- |
| Dependent Variable: WBCs% | | | | | |
| Constant | 5.44 | 3.458 | 7.422 | 1.005 | <0.001* |
| Time to reperfusion (T4-8 vs T4/T8) | -1.602 | -2.451 | -0.753 | 0.431 | <0.001* |
| Thrombolytic treatment | 0.600 | -0.273 | 1.473 | 0.443 | 0.177 |
| Atrial fibrillation | 0.044 | -0.815 | 0.904 | 0.436 | 0.919 |
| Stroke cause (TOAST) | 0.587 | -0.315 | 1.489 | 0.458 | 0.201 |

# Data Set

The detailed statistics were applied in the supplement.

**Figure 1**. RBCs, fibrin/platelets and WBCs in thrombi in relation to the different durations of time to reperfusion. (A) Representative image of thrombi sections stained with Martius Scarlett Blue comparing <4 h (T4), 4–8 h (T4–8) and >8 h (T8) time to reperfusion demonstrating the presence of RBCs (yellow), fibrin/platelets (red or purple) and WBCs (blue). Low-magnification scale bar = 1 mm; High-magnification scale bar = 60 μm. (B) Boxplot indicating thrombi composition changes with different times to reperfusion.

Figure 1 (B) WBCs changes with different times to reperfusion.

|  | T4 | T4-8 | T8 | F (df1, df2) =  value | *P* |
| --- | --- | --- | --- | --- | --- |
| Median (IQR) | 3.050(1.953-4.630) | 4.218 (2.393-6.033) | 2.424 (1.197-4.651) |  |  |
| Normality ： Shapiro-Wilk normality test (W) P | 0.944(0.049) | 0.867(<0.001) | 0.903(0.001) |  |  |
| Homogeneity of variances test: Levene Statistic |  |  |  | F(2,203)=3.891 | 0.022 |

|  | T4-8 vs. T4 (P) | T4-8 vs. T8 (P) | Test statistic | *P* |
| --- | --- | --- | --- | --- |
| One way ANOVA |  |  | 7.920 | <0.001 |
| Dunnett’s T3 test | 0.002 | 0.001 |  |  |

**Figure 2**. Neutrophil and NET changes in thrombi at different time after reperfusion. (A) Representative immunohistochemical illustration of neutrophils (MPO, red) in thrombi of the T4, T4–8 and T8 groups. Nuclei are counterstained with DAPI. Scale bar = 50 μm. (B) Quantification of MPO+ cells/mm^2^ (top), WBCs/mm^2^ (middle) and MPO% (bottom) in thrombi according to different times to reperfusion. n=20/group. (C) Representative immunohistochemical illustration of NETs in thrombi by staining for MPO (red) and Cit3H4 (green). Nuclei are counterstained with DAPI. Scale bar = 50 μm. (D**)** Quantification of NETs/mm^2^ in thrombi according to time to reperfusion. T4, n=20; T4–8, n=17; T8, n=17. NETs=neutrophil extracellular traps.

Figure 2 (B) Quantification of MPO+ cells/mm^2^ in thrombi according to time to reperfusion.

|  | T4 | T4-8 | T8 | F (df1, df2) =  value | *P* |
| --- | --- | --- | --- | --- | --- |
| Median (IQR) | 207.917(155.208-408.958) | 462.917 (303.333-718.125) | 225.417 (107.083-398.750 |  |  |
| Normality ： Shapiro-Wilk normality test (W) P | 0.888(0.024) | 0.897(0.036) | 0.925(0.125) |  |  |
| Homogeneity of variances test: Levene Statistic |  |  |  | F(2,57)=2.168 | 0.124 |

|  | T4-8 vs. T4 (P) | T4-8 vs. T8 (P) | Test statistic | *P* |
| --- | --- | --- | --- | --- |
| Kruskal-Wallis test |  |  | 15.247 | <0.001 |
| Kruskal-Wallis one-way ANOVA followed by Bonferroni’s correction | 0.003 | 0.001 |  |  |

Figure 2 (B) Quantification of WBCs/mm^2^ in thrombi according to time to reperfusion.

|  | T4 | T4-8 | T8 | F (df1, df2) =  value | *P* |
| --- | --- | --- | --- | --- | --- |
| Median (IQR) | 623.333(384.792-836.250) | 976.875 (685.417-1223.542) | 602.083 (331.458-864.375) |  |  |
| Normality ： Shapiro-Wilk normality test (W) P | 0.899(0.039) | 0.941(0.246) | 0.946(0.314) |  |  |
| Homogeneity of variances test: Levene Statistic |  |  |  | F(2,57)=0.087 | 0.917 |

|  | T4-8 vs. T4 (P) | T4-8 vs. T8 (P) | Test statistic | *P* |
| --- | --- | --- | --- | --- |
| One way ANOVA |  |  | 8.133 | 0.001 |
| Bonferroni’s  multiple  comparisins test | 0.005 | 0.002 |  |  |

Figure 2 (D) Quantification of NETs/mm^2^ in thrombi according to time to reperfusion.

|  | T4 | T4-8 | T8 | F (df1, df2) =  value | *P* |
| --- | --- | --- | --- | --- | --- |
| Median (IQR) | 43.333(12.500-134.375) | 147.500 (27.083-203.333) | 45.833 (3.472-63.889) |  |  |
| Normality ： Shapiro-Wilk normality test (W) P | 0.737(<0.001) | 0.891(0.049) | 0.871(0.023) |  |  |
| Homogeneity of variances test: Levene Statistic |  |  |  | F(2,51)=3.769 | 0.030 |

|  | T4-8 vs. T8 (P) | Test statistic | *P* |
| --- | --- | --- | --- |
| Kruskal-Wallis test |  | 6.421 | 0.040 |
| Kruskal-Wallis one-way ANOVA followed by Bonferroni’s correction | 0.041 |  |  |

**Figure 3**. Association of NETs in thrombi with clinical features. (A) Correlation between WBCs in thrombi and WBCs in peripheral blood. (B) Correlation between neutrophils in thrombi and neutrophils in peripheral blood. (C) Correlation between NIHSS score (pre) and NETs in thrombi. (D) Correlation between NIHSS score (post) and NETs in thrombi. (E) Forest plot of multivariate logistic regression analysis for patients with AIS including possible outcome influencing factors regarding the correlation analysis. AIS= acute ischemic stroke; NIHSS= National Institutes of Health Stroke Scale; mRS= modified Rankin Scale.

Figure 3 (E) Multivariate logistic regression analysis for patients with AIS including possible outcome influencing factors regarding the correlation analysis.

|  | mRS 0-2  (n=109) | mRS ≥3  (n=97) | *P* |
| --- | --- | --- | --- |
| Age; Median (IQR) | 71.0(63.0-78.0) | 77.0(66.5-83.5) | <0.001* |
| Male (%) | 57(52.3) | 37(38.1) | 0.042* |
| Hypertension | 50(45.9) | 57(58.8) | 0.065 |
| Diabetes | 18(16.5) | 19(19.6) | 0.566 |
| Hyperlipidemia | 14(12.8) | 8(8.2) | 0.286 |
| Coronary artery disease | 32(29.4) | 42(43.3) | 0.037* |
| Active smoker | 17(15.6) | 9(9.3) | 0.173 |
| Atrial fibrillation | 51(46.8) | 61(62.9) | 0.021* |
| Time to reperfusion, min | 320.0(259.5-432.5-) | 330.0(275.0-447.5) | 0.541 |
| Occlusion site (%) |  |  |  |
| Anterior circulation | 94(86.2) | 90(92.8) | 0.129 |
| Posterior circulation | 15(13.8) | 7(7.2) | 0.129 |
| Stroke cause (TOAST) (%) |  |  |  |
| Arterioembolic | 26(23.9) | 12(12.4) | 0.034* |
| Cardioembolic | 74(67.9) | 81(83.5) | 0.010* |
| Cryptogenic | 9(8.3) | 4(4.1) | 0.223 |
| Antithrombotic treatment (%) | 14(12.8) | 13(13.4) | 0.906 |
| Thrombolytic treatment (%) | 43(39.4) | 28(28.9) | 0.111 |
| Procedural technique (%) |  |  |  |
| Stentriever | 105(96.3) | 91(93.8) | 0.402 |
| Direct aspiration | 3(2.8) | 4(4.1) | 0.588 |
| Arterial thrombolysis | 17(15.6) | 21(21.6) | 0.264 |
| Balloon angioplasty | 10(9.2) | 5(5.2) | 0.268 |
| ADAPT | 3(2.8) | 2(2.1) | 0.748 |
| Stent placement | 10(9.2) | 5(5.2) | 0.268 |
| Procedure time, min | 75.0(55.0-106.0) | 90.0(56.0-140.0) | 0.021* |
| No. of maneuvers | 1.0(1.0-2.0) | 2.0(1.0-2.0) | 0.557 |
| mTICI score 2b-3 (%) | 106(97.2) | 92(94.8) | 0.373 |
| NIHSS score (pre) | 18.0±40.8 | 21.0±35.7 | 0.001* |
| NIHSS score (post) | 3.0(1.0-7.0) | 12.0(7.0-17.0) | <0.001* |
| RBCs (%) | 65.1(31.3-86.9) | 50.8(26.2-85.9) | 0.155 |
| Fibrin/platelets (%) | 31.4(9.6-62.0) | 41.7(9.6-69.6) | 0.170 |
| WBCs (%) | 3.4(1.8-5.3) | 3.9(2.3-5.8) | 0.957 |

**Figure 4**. Association of NETs in thrombi with collateral flow of patients with ischemic stroke. (A) Examples of collateral flow with DSA-based collateral grades. All DSAs images show proximal middle cerebral artery (MCA) occlusions (arrows). DSA consists of arterial (left panel) and venous phases (right panel). Poor grade: no collaterals visible or slow collaterals visible only in the late phase. Intermediate grade: rapid collateral flow to part of the occluded MCA territory with persistence of some of the defect. Good grade: slow but complete collateral flow in the occluded MCA territory. Excellent grade: rapid and complete collateral flow in the occluded MCA territory. (B) Distribution of collateral grade in patients with different times to reperfusion. (C) Quantification of NETs/mm^2^ in the thrombi of patients with poor (n=13), intermediate-to-good (n=14) and excellent (n=21) collateral flows. NETs=neutrophil extracellular traps.

Figure 4 (C) Quantification of NETs/mm^2^ in the thrombi of patients with different collateral grades.

|  | T4 | T4-8 | T8 | F (df1, df2) =  value | *P* |
| --- | --- | --- | --- | --- | --- |
| Median (IQR) | 43.333(12.500-134.375) | 147.500 (27.083-203.333) | 45.833 (3.472-63.889) |  |  |
| Normality ： Shapiro-Wilk normality test (W) P | 0.886(0.086) | 0.867(0.038) | 0.793(0.001) |  |  |
| Homogeneity of variances test: Levene Statistic |  |  |  | F(2,45)=4.898 | 0.012 |

|  | Excellent vs. Poor (P) | Excellent vs. Intermediate-Good (P) | Test statistic | *P* |
| --- | --- | --- | --- | --- |
| Kruskal-Wallis test |  |  | 8.012 | 0.018 |
| Kruskal-Wallis one-way ANOVA followed by Bonferroni’s correction | 0.039 | 0.079 |  |  |
